# Supplementary material for: Quantitative proteome of bacterial periplasmic predation by Bdellovibrio bacteriovorus reveals a prey-lytic protease
Source: Commun Biol. 2025 Oct 22;8:1491. doi: 10.1038/s42003-025-09010-x (PMC12546842; doi:10.1038/s42003-025-09010-x)
Supplement: Supplementary file 1 — Supplementary Information [file 42003_2025_9010_MOESM1_ESM.pdf]

Supplementary Information

Quantitative proteome of bacterial periplasmic predation by *Bdellovibrio bacteriovorus* reveals a prey-lytic protease

Ting F. Lai[1], Denis Jankov[1], Jonas Grossmann[2,3], Bernd Roschitzki[2], Simona G. Huwiler[1,#]

[1]Department of Plant and Microbial Biology, University of Zurich, Zurich, Switzerland

[2]Functional Genomics Center Zurich, University of Zurich/ETH Zurich, Zurich, Switzerland

[3]Swiss Institute of Bioinformatics (SIB) Quartier Sorge - Batiment Amphipole, 1015 Lausanne, Switzerland

Correspondence: Simona G. Huwiler, simona.huwiler@uzh.ch

Content:

Supplementary Figures 1-6	page 2 - 7
Supplementary Data 1-3 legends	page 8
Supplementary Data 1-3	available online in Supplementary Data
Supplementary Tables 1-4	page 9 - 12
Supplementary References	page 13

Supplementary Figures

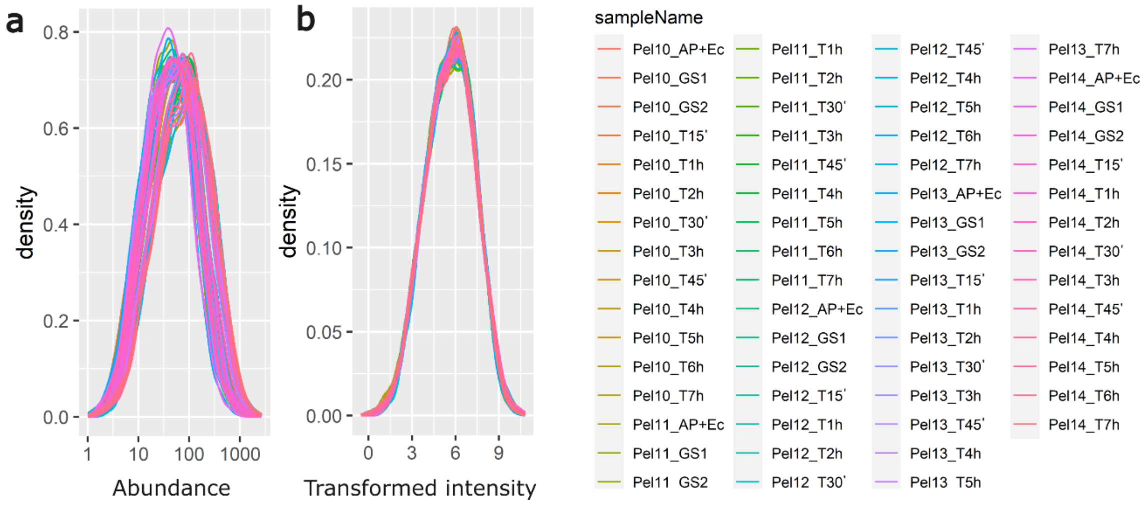

Supplementary Fig. 1: Density plots showing the effect of sample normalization. a, Before and **b,** after scaling of protein abundance by internal reference scaling with robust scaling normalizations of all samples of the five biological repeats (Pel10-Pel14). This normalization method takes geometric mean for each protein, calculates scaling factors for each protein to adjust the protein abundance to the geometric mean. The 'AP+Ec' condition combines the protein abundances from the 'attack phase (AP)' containing *Bdellovibrio* only' and '*E. coli* only (Ec)', for better comparison with other timepoints. Golden standard samples (GS1, GS2) comprise an equal mixture from each condition and TMT set.

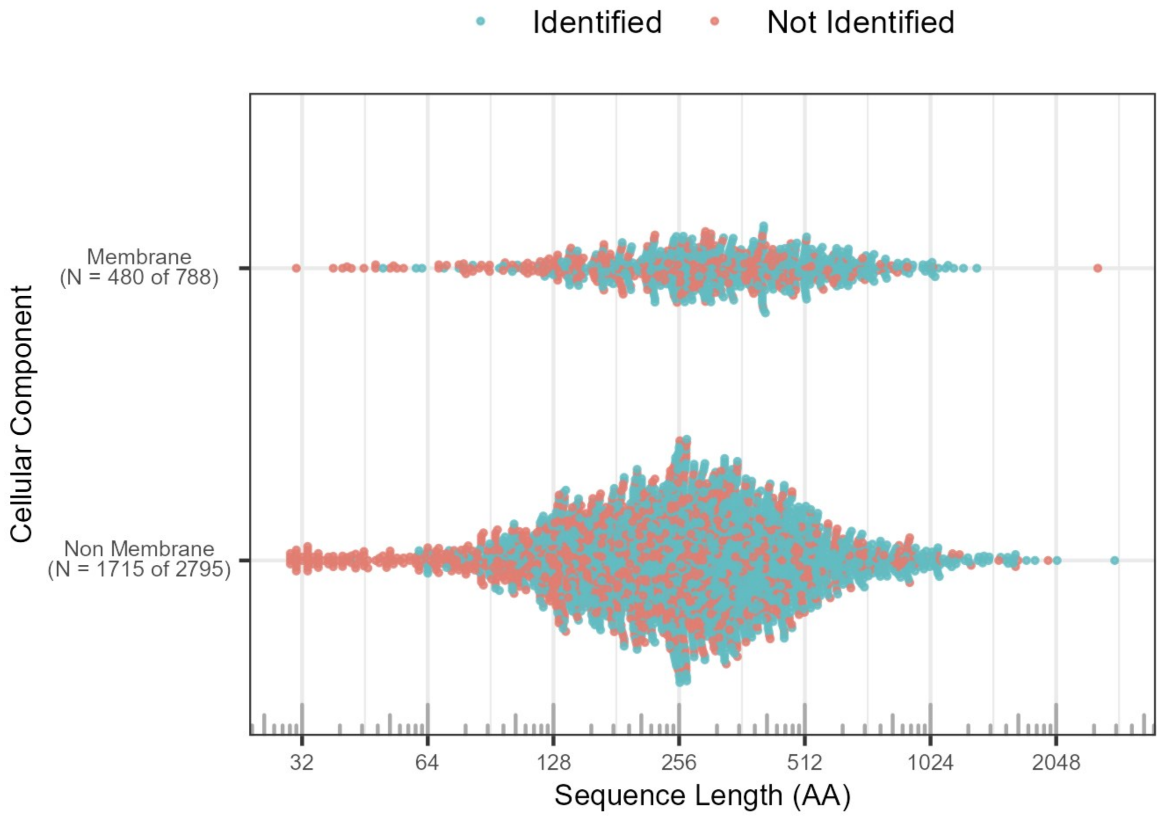

Supplementary Fig. 2: Distribution plot of membrane and non-membrane proteins in the quantitative proteome *of B. bacteriovorus*, and their identification status. The plot shows the protein length distribution in amino acids (AA) on a $\log_2$ scale and whether proteins were identified (turquoise dots) or not identified (red dots). The ratio of membrane and non-membrane proteins identified in the quantitative proteome very closely matched those predicted from the *B. bacteriovorus* reference proteome (22% membrane vs. 78% non-membrane). N indicates the absolute number of proteins that were identified in this quantitative proteome of the ones in the database.

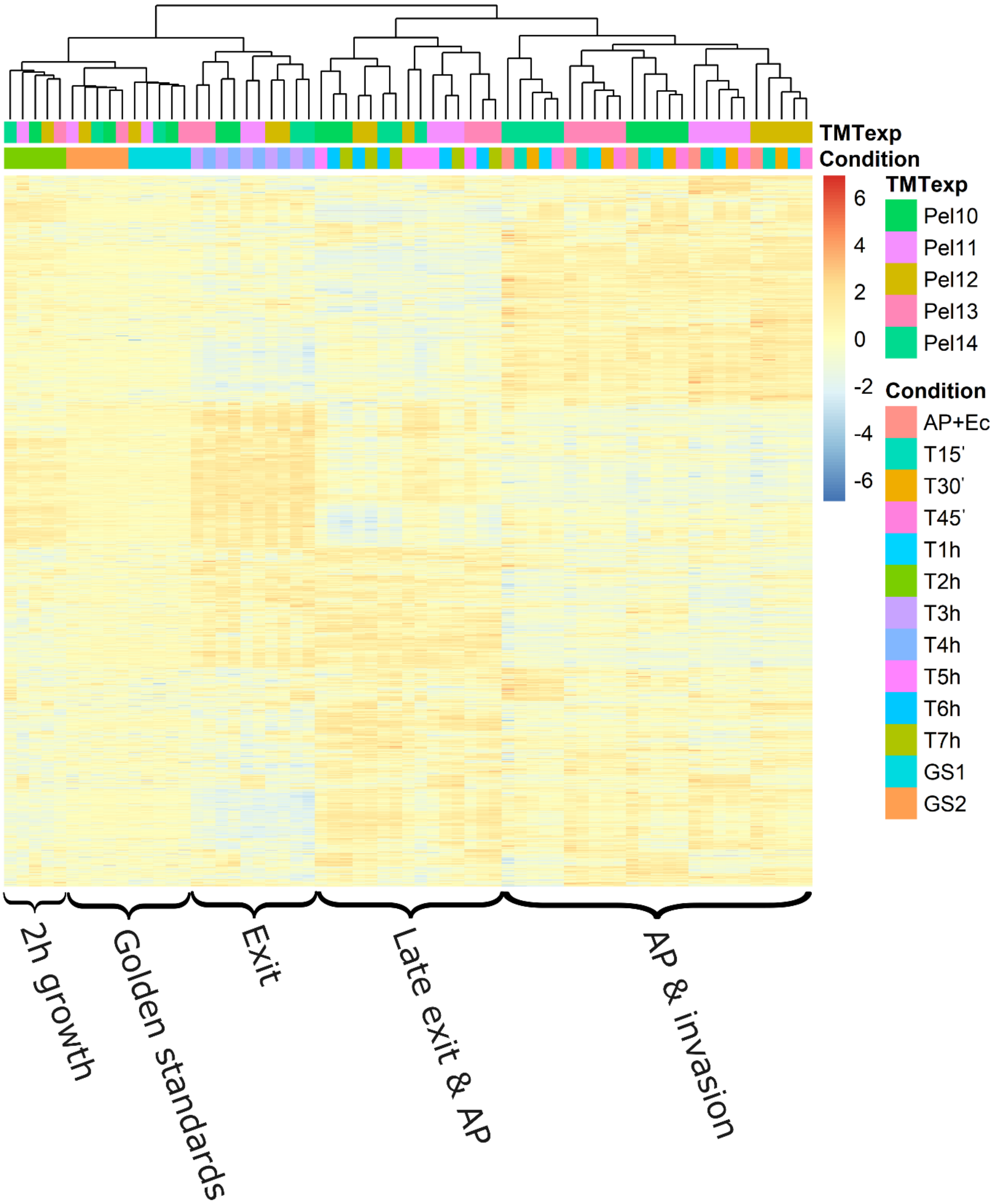

Supplementary Fig. 3: Hierarchically clustered heatmap showing the relationship between samples with regards to the five biological repeats and the condition. Proteins were grouped based on the five biological repeats processed in separate TMT sets (TMTexp) and the sampling timepoints of the predatory life cycle (Condition). The 'Condition' timepoints are further grouped categorized by their corresponding life cycle phases, as indicated at the bottom of the heatmap. The units are of relative internal reference scaled intensity of protein abundance from the normalized data. For abbreviations, please refer to Supplementary Figure 1. The golden standard samples (GS1, GS2) cluster together, indicating effective normalization.

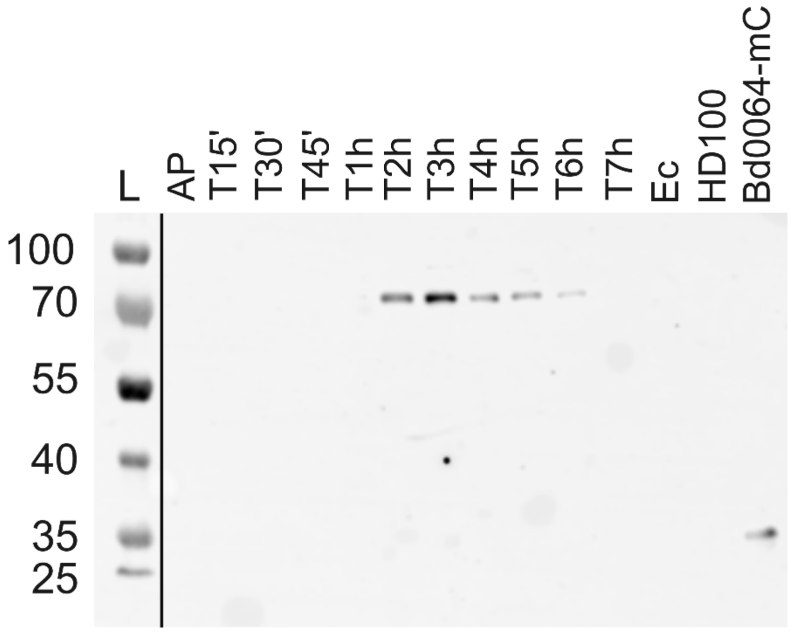

Supplementary Fig. 4: Western blot of Bd2269-mCherry expression over the whole predatory life cycle. Semi-quantitative analysis reveals Bd2269 to be most expressed at 3 hours corroborating our quantitative proteome data. The expected size of Bd2269-mCherry is ~83 kDa, while positive control Bd0064-mCherry is ~40.5 kDa. We speculate that the slightly lighter weight band of Bd0064mCherry could be due to partial degradation. AP = 'attack phase' *B. bacteriovorus* HD100 cells; T15'-7h = timepoints in minutes then hours after invasion; Ec = *E. coli* K-12 pZMR100 prey; HD100 = *B. bacteriovorus* HD100 wild-type control; Bd0064-mC = Bd0064-mCherry positive control[1]; L = Protein ladder (kDa) is separated from the Western blot signals by a line to show that it is not chemiluminescent. Two independent biological repeats were performed with the most representative result shown here. The blot of the second independent biological repeat, including its uncropped image files can be found in the FigShare folder. The uncropped original image of this Supplementary Figure 4 can be found in Supplementary Figure 6.

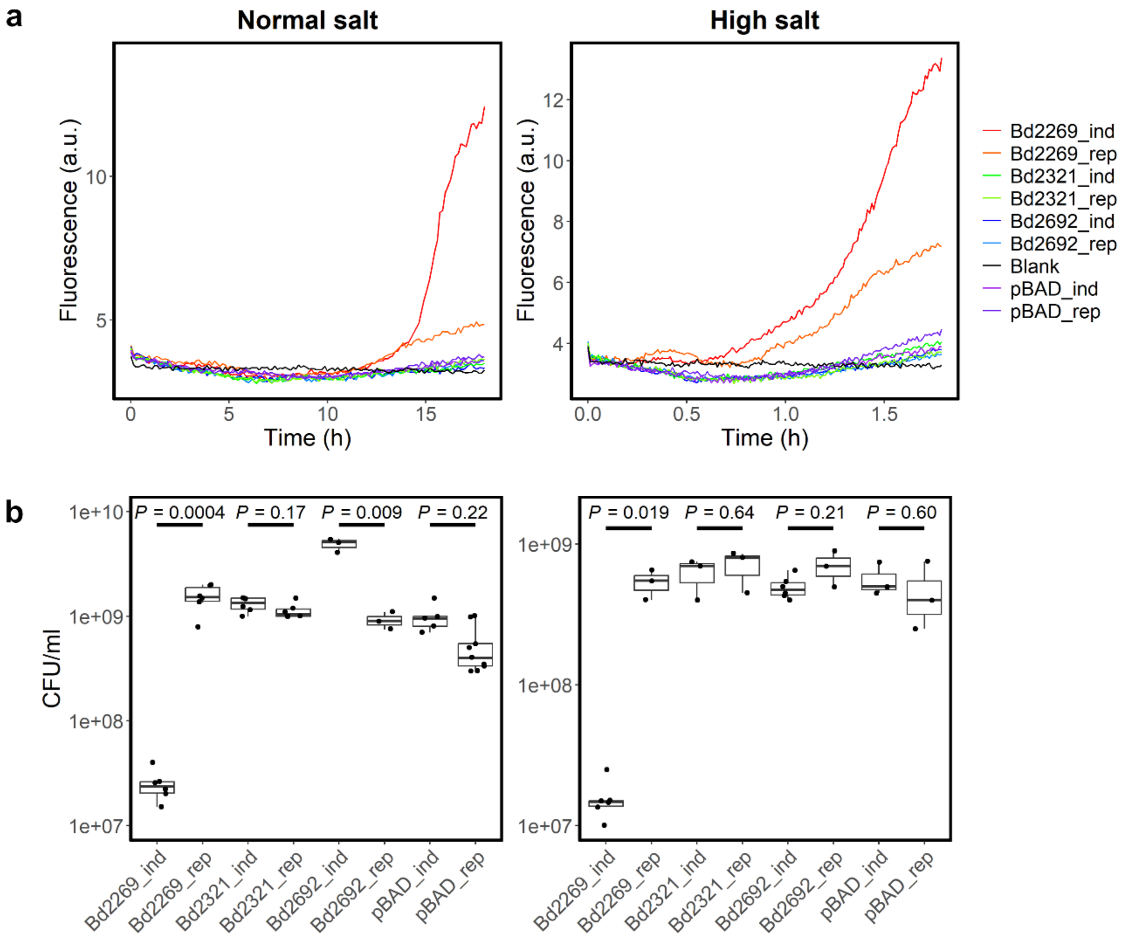

Supplementary Fig. 5. Second repeat of heterologous expression of protease Bd2269 in *E. coli* S17-1 (see Fig. 4bc). a, *E. coli* S17-1 damage from proteases Bd2269, Bd2321 and Bd2692 was tested by β-galactosidase activity assay measuring the fluorescence of chlorophenol red under normal (0.171 M NaCl) and high (0.4 M NaCl) salt conditions over 18 hours. Bd2269, Bd2321 and Bd2692 were heterologously expressed in *E. coli* S17-1 from a pBAD18-based plasmid under inducing ('_ind') or repressing ('_rep') conditions. 'pBAD' stands for the empty vector. *E. coli* S17-1 overexpressing Bd2269 (Bd2269_ind) caused self-lysis and β-galactosidase release over time. **b,** Boxplots showing *E. coli* cell viability 18 hours after the start of protease induction ('_ind') or repression ('_rep') in different protease expression strains (Bd2269, Bd2321, Bd2692, pBAD [empty vector control]). The median and interquartile range (IQR) were plotted, represented by the box. The whiskers extend to 1.5 times the IQR and black dots outside of this range are outliers. Two-tailed Welch two-sample t-tests compare the mean values between the induced and repressed conditions for each protease (*p*-values indicated in graph). Three technical repeats were evaluated across a dilution range of 4.

Rep1 overlay

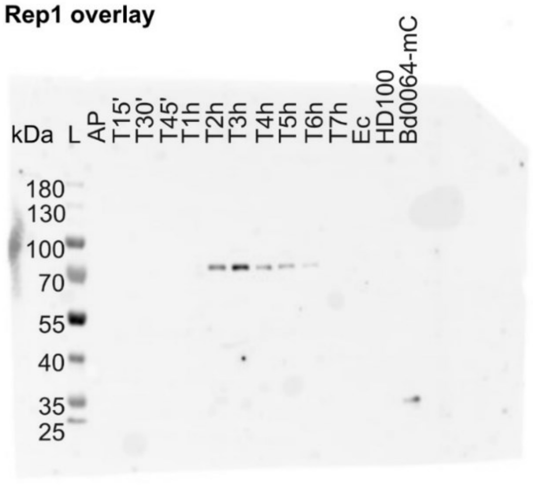

Rep1 chemi only

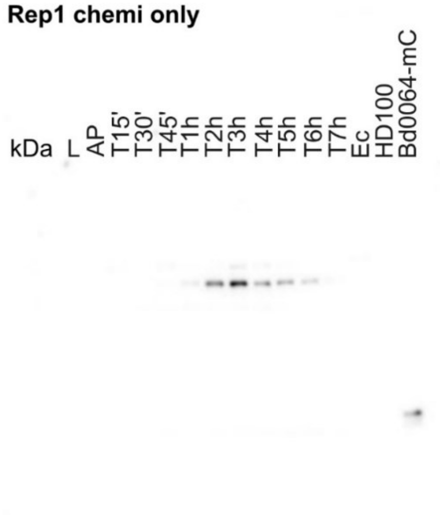

Rep1 ladder

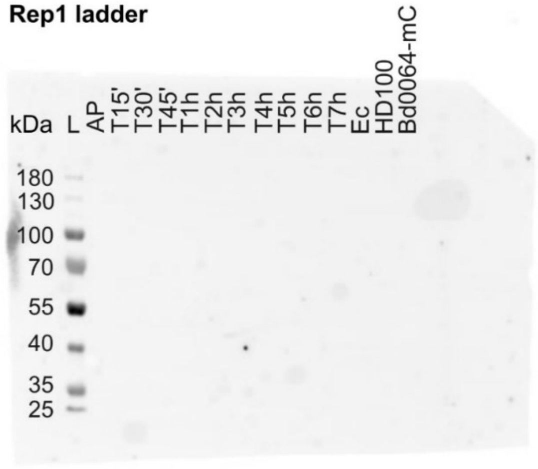

Supplementary Fig. 6: Uncropped raw image data of Western blot of Bd2269-mCherry expression over the whole predatory life cycle shown in Supplementary Figure 4. The latter is an overlay of the chemiluminescence channel and the visible channel to detect the marker lane.

Supplementary Data

For Supplementary Data 1-3 there are Data legends only in this document, as the Table data itself is available online as .xls files.

Supplementary Data 1: A table containing all normalized protein abundances of each protein over a 7-hour period. Protein abundances are normalized by internal reference scaling with TMM and robust scaling (see methods). Displayed are protein accessions, names, NCBI descriptions, detailed EGGnog descriptions[2], with e-values and the number of unique peptides mapped to each protein.

Supplementary Data 2: Tables with proteins grouped into the nine clusters of distinct abundance patterns throughout the predatory life cycle. Each cluster is named as per Fig. 2b. The table includes manually combined cluster numbers and lists the protein accessions for each cluster.

Supplementary Data 3: Tables containing all the log_2 fold change values of *B. bacteriovorus* HD100 proteins between each condition and attack phase. The different contrasts are separated into their respective tabs. Displayed in the column headers are: protein accessions, names, NCBI descriptions, EGGnog descriptions[2] ("-" = No annotation), with e-values (#N/A = no e-value). Further shown are: contrast comparing the different condition to attack phase, log_2 (fold change, FC), adjusted p-value (FDR), the number of unique peptides mapped to each protein (#Unique peptides), significance based on FDR < 0.05 (significant005), and an indication of whether the protein was experimentally determined or imputed (Imputed?). The log_2(FC) values here were used to plot Fig. 3.

Supplementary Tables

Supplementary Table 1: Primers used in this study.

Primer name	Sequence (5' to 3')	Description
2269_EcoRI_F	agcttggaattcatgaaattcaacgtgtttgc	Construction of pBAD18-Bd2269. Addition of EcoRI and SalI restriction sites.
2269_SalI_R	tacgttgtcgacttacttcgcgtcgatcgcag	
2692_EcoRI_F	agcttggaattcatgaaacgtgcattactagt	Construction of pBAD18-Bd2692. Addition of EcoRI and SalI restriction sites.
2692_SalI_R	tacgttgtcgacctactgacagaatggtaggt	
2321_GIB_F	ccatacccgtttttttgggctagcgatgaatcatcttgtgaagggc	Construction of pBAD18-Bd2321 plasmid. Addition of overhangs for Gibson assembly.
2321_GIB_R	acagccaagcttgcatgcctgcaggctatttagccgtataaactagagc	
pBAD_F	cacggcagaaaagtccacat	Sequencing of pBAD18-protease constructs.
pBAD_R	ctctcatccgccaaaacagc	
bd2269KO_up_F	attcacgatacctttcattacaattccccccatatccatgg	Construction of pK18-Δ*bd2269*. Amplification of 1-kb upstream region.
bd2269KO_up_R	ggaaacagctatgaccatgattacgtgcagcggctgcgtgagc	
bd2269KO_down_F	cgttgtaaaacgacggccagtgccatcttgatcggagcaatcag	Construction of pK18-Δ*bd2269*. Amplification of 1-kb downstream region.
bd2269KO_down_R	aattgtaatgaaaggtatcgtgaatgcggaag	
bd2269KO_seq_F	cagcatcccgaccgagataa	Sequencing of pK18-Δ*bd2269*.
bd2269KO_seq_R	ggtcattcccgcttacaacg	
Bd2269Native-F	cctgcaggtcgactgactgattgacccgctctggtcctaaaatcgaac	Construction pCAT-*bd2269* for complementation. Amplification of *bd2269* and 200bp upstream promoter region. Addition of overhangs for Gibson assembly with pCAT backbone of pFL021.
Bd2269Native-R	cgtcgtccttgtagtctcctgctcccttcgcgtcgatcgcagcttg	
PCATBackbone-F	taattgactgaagtccactggc	Amplification of pCAT plasmid backbone of pFL021.
PCATBackbone-R	cctgcaggcatgcaagcttg	
PCAT-F	tgccacctgacgtctaagaa	Sequencing of pCAT-*bd2269*
PCAT-R	tggcttaactatgcggcatc	
PCAT-M	ggtcttatgcgtctaag	
2269_3p_1kb_F	cgttgtaaaacgacggccagtgccattcactggtgttgctcctaaag	Construction of pK18-Bd2269:mCherry. Amplification of Bd2269 1kb region from 3' end with overlap regions for Gibson assembly.
2269_3p_1kb_R	cttgctcaccatcttcgcgtcgatcgcagc	
mCherry_2269_F	gatcgacgcgaagatggtgagcaagggcgag	Construction of pK18-Bd2269:mCherry. Amplification of mCherry with overlap regions for Gibson assembly.
mCherry_pK18_R	ggaaacagctatgaccatgattacgctacttgtacagctcgtccatg	

Supplementary Table 2: Plasmids used in this study. Amp^R = ampicillin resistance, Kan^R = kanamycin resistance, MCS = multiple cloning site.

Plasmid	Description	Source
pBAD18	Arabinose-inducible plasmid (*araBAD* promoter), Kan^R, used to express *B. bacteriovorus* proteases in *E. coli*.	Gift from Leo Eberl. Guzman *et al*., 1995[3].
pBAD18-*bd2269*	*bd2269* cloned into MCS of pBAD18 for arabinose induction.	This study
pBAD18-*bd2321*	*bd2321* cloned into pBAD18 by Gibson assembly for arabinose induction.	This study
pBAD18-*bd2692*	*bd2692* cloned into MCS of pBAD18 for arabinose induction	This study
pK18*mobsacB*	Suicide vector (Kan^R, *lacZα*, *sacB*) used for crossovers into the *B. bacteriovorus* genome.	Gift from Prof. R. E. Sockett. Schäfer *et al*., 1994[4].
pK18-Δ*bd2269*	1-kb upstream and downstream regions of *bd2269* in pK18mobsacB to make bd2269 marker-less gene deletion.	This study
pCAT.000	Self replication level T acceptor vector with *lacZ*. Modified BioBrick vector, from RSF1010: ori, RepB and RepC; Kan^R, Amp^R,	From Addgene (plasmid #119559). Vasudevan *et al*., 2019[5].
pFL015 (pCAT-based)	pCAT.000-PmerRNA-RBS-mCherry. Amplification of the pCAT backbone for expression within *B. bacteriovorus*. (Plasmid also contained promotor of merRNA[6], an optimized RBS for B. *bacteriovorus*[7], and an mCherry sequence not directly used in the project. This plasmid was used as template for backbone of pFL021.	Generated by and gift from Florian Lindner.
pFL021 (pCAT-based)	pCAT.000-P_{merRNA}-RBS-VirF-FLAG. This plasmid is based on pFL015 and was used as template for backbone amplification to include the sequence of a C-terminal FLAG-tag.	Generated by and gift from Florian Lindner.
pCAT-*bd2269*	pCAT plasmid containing 196-pb of upstream region of *bd2269* (including natural promoter region), followed by *bd2269* with an additional sequence for a C-terminal FLAG tag (amplified from pFL021). This plasmid was used for complementation of *Δbd2269* to measure exit speed (Fig. 4a).	This study
pK18-*bd2269*:*mCherry*	pK18*mobsacB* containing sequence for Bd2269 fused with mCherry at the C-terminus (single crossover) for homologous recombination in *B. bacteriovorus*. Used for Western blot confirmation (Supplementary Fig 4).	This study

Supplementary Table 3: Strains used in this study. Kan^R = kanamycin resistance.

Strain	Description	Source
E. coli NEB5α	*E. coli* cloning strain (*fhuA2Δ(argF-lacZ)U169 phoA glnV44 Φ80Δ(lacZ)M15 gyrA96 recA1 relA1 endA1 thi-1 hsdR17)*	New England Biolabs (C2987)[8].
E. coli S17-1	*E. coli* strain (*thi, pro, hsdR-, hsdM+, recA*; integrated plasmid RP4- Tc::Mu-Kn::tn)	Gift from Prof. R. E. Sockett, University of Nottingham.<br>Lambert *et al*., 2006[9] & Simon *et al*., 1983[10].
E. coli S17-1 pZMR100	*E. coli* S17-1 strain containing the plasmid pZMR100 (Kan^R), used as prey for kanamycin-resistant *B. bacteriovorus.*	Gift from Prof. R. E. Sockett, University of Nottingham.<br>Lambert et al. 2006[9] & Rogers *et al*., 1986[11].
E. coli S17-1 pBAD18	*E. coli* S17-1 strain used as a negative control for arabinose-protease induction assay containing no protease gene after araBAD promoter	This study
E. coli S17-1 pBAD18-*bd2269*	*E. coli* S17-1 strain expressing Bd2269 when induced with arabinose.	This study
E. coli S17-1 pBAD18-*bd2321*	*E. coli* S17-1 strain expressing Bd2321 when induced with arabinose.	This study
E. coli S17-1 pBAD18::*bd2692*	*E. coli* S17-1 strain expressing Bd2692 when induced with arabinose.	This study
E. coli S17-1 pMAL-p2_mCherry	*E. coli* S17-1 strain expressing a maltose binding protein-mCherry fusion with a *malE* signal sequence, directing it to the periplasm. This provides an all fluorescent background to detect division of non-fluorescent *B. bacteriovorus* by fluorescence microscopy.	Gift from Prof. R. E. Sockett, University of Nottingham. Fenton *et al*., 2010[12].
E. coli K-12 MG1655	Model *E. coli* strain used as prey for *B. bacteriovorus* predation assays.	Blattner *et al*., 1997[13].
E. coli K-12 MG1655 pZMR100	*E. coli* K-12 MG1655 strain containing pZMR100 plasmid which confers Kan^R. Used as prey for Kan^R *B. bacteriovorus* strains.	This study
E. coli NEB5α pK18-*bd2269*:mCherry	Assembly of pK18-*bd2269*:mCherry	This study
E. coli S17-1 pK18-*bd2269*:mCherry	Donor strain for conjugation into wild-type *B. bacteriovorus* HD100 for Western blot verification	This study
E. coli NEB5α pK18-Δ*bd2269*	Assembly of pK18-Δ*bd2269*	This study
E. coli S17-1 pK18-Δ*bd2269*	Donor strain for conjugation into wild-type *B. bacteriovorus* HD100 and separately *B. bacteriovorus* Δ*bd0314* to delete *bd2269*	This study
E. coli NEB5α pCAT-*bd2269*	Assembly of pCAT-*bd2269*	This study
E. coli S17-1 pCAT-*bd2269*	Donor strain for conjugation into *B. bacteriovorus* Δ*bd2269* for complementation	This study

B. bacteriovorus HD100[T]	*B. bacteriovorus* Type strain, wild-type	Gift from Prof. R. E. Sockett, University of Nottingham. Rendulic *et al.*, 2004[14].
B. bacteriovorus HD100 *bd2269*::*mCherry*	*B. bacteriovorus* HD100 with *bd2269*::*mCherry* integrated into genome by single crossover recombination. Used for Western blots confirmation of Bd2269 protein level.	This study
B. bacteriovorus HD100 *bd0064:mCherry*	*B. bacteriovorus* HD100 with *bd0064*:*mCherry* integrated into genome by single crossover recombination. Used as a positive control for Western blots confirmation.	Gift from Prof. R. E. Sockett[1,] University of Nottingham.
B. bacteriovorus HD100 Δ*bd2269*	*B. bacteriovorus* HD100 with a clean deletion of *bd2269,* used for microscopy exit time analysis.	This study
B. bacteriovorus HD100 Δ*bd2269* pCAT-*bd2269*	*B. bacteriovorus HD100 Δbd2269* with pCAT-*bd2269* for complementation of *Δbd2269*	This study
B. bacteriovorus HD100 Δ*bd0314*	*B. bacteriovorus* HD100 with a clean deletion of *bd0314* for microscopy exit time analysis	Gift from Prof. R. E. Sockett, University of Nottingham. Harding *et al*., 2020[15].
B. bacteriovorus HD100 Δ*bd2269* Δ*bd0314*	*B. bacteriovorus* HD100 with a clean deletion of *bd2269* and *bd0314* for microscopy exit time analysis.	This study

Supplementary Table 4: Sample size estimation for a statistical power of 0.8 and a significance level of 0.05, for samples generated from the attack and growth phases of the predatory life cycle of *B. bacteriovorus*. Sample size requirements for attack phase (AP) and growth phase at 1 hour (T1h) conditions were estimated for fold change (FC) thresholds of >1.5, 2 or 4. Lower FC thresholds require more repeats due to increased detail in protein information. The number of biological repeats needed for total protein proportions achieving a statistical power of 0.8 is shown, ranging from 50% to 90%. Highlighted in yellow are the sample numbers required for 80% of proteins to achieve a power of 0.8 at each FC threshold listed in the table.

**Proportion of total proteins (%)**	**Condition**	**No. of samples required**		
		**FC > 1.5**	**FC > 2**	**FC > 4**
50	AP	5	3	2
50	T1h	3	3	2
70	AP	9	4	3
70	T1h	4	3	2
80	AP	12	5	3
80	T1h	5	3	2
90	AP	22	9	4
90	T1h	7	4	2

Supplementary References

1. Willis, A. R. *et al.* Injections of predatory bacteria work alongside host immune cells to treat *Shigella* infection in zebrafish larvae. *Current Biology* **26**, 3343–3351 (2016).

2. Cantalapiedra, C. P., Hernández-Plaza, A., Letunic, I., Bork, P. & Huerta-Cepas, J. eggNOG-mapper v2: functional annotation, orthology assignments, and domain prediction at the metagenomic scale. *Molecular Biology and Evolution* **38**, 5825–5829 (2021).

3. Guzman, L. M., Belin, D., Carson, M. J. & Beckwith, J. Tight regulation, modulation, and high-level expression by vectors containing the arabinose PBAD promoter. *J Bacteriol* **177**, 4121–4130 (1995).

4. Schäfer, A. *et al.* Small mobilizable multi-purpose cloning vectors derived from the *Escherichia coli* plasmids pK18 and pK19: selection of defined deletions in the chromosome of *Corynebacterium glutamicum*. *Gene* **145**, 69–73 (1994).

5. Vasudevan, R. *et al.* CyanoGate: A modular cloning suite for engineering Cyanobacteria based on the plant MoClo syntax. *Plant Physiol.* **180**, 39–55 (2019).

6. Karunker, I., Rotem, O., Dori-Bachash, M., Jurkevitch, E. & Sorek, R. A global transcriptional switch between the attack and growth forms of *Bdellovibrio bacteriovorus*. *PLoS ONE* **8**, e61850 (2013).

7. Dwidar, M. & Yokobayashi, Y. Controlling *Bdellovibrio bacteriovorus* gene expression and predation using synthetic riboswitches. *ACS Synth. Biol.* **6**, 2035–2041 (2017).

8. Anton, B. P. & Raleigh, E. A. Complete genome sequence of NEB 5- α, a derivative of *Escherichia coli* K-12 DH5α. *Genome Announc* **4**, e01245-16 (2016).

9. Lambert, C. *et al.* Characterizing the flagellar filament and the role of motility in bacterial prey-penetration by *Bdellovibrio bacteriovorus*. *Mol Microbiol* **60**, 274–286 (2006).

10. Simon, R., Priefer, U. & Puhler, A. A broad host range mobilization system for in vivo genetic engineering: transposon mutagenesis in gram negative bacteria. *Bio/Technology* **1**, 784–791 (1983).

11. Rogers, M., Ekaterinaki, N., Nimmo, E. & Sherratt, D. Analysis of Tn7 transposition. *Mol Gen Genet* **205**, 550–556 (1986).

12. Fenton, A. K., Kanna, M., Woods, R. D., Aizawa, S.-I. & Sockett, R. E. Shadowing the actions of a predator: backlit fluorescent microscopy reveals synchronous nonbinary septation of predatory *Bdellovibrio* inside prey and exit through discrete Bdelloplast pores. *Journal of Bacteriology* **192**, 6329–6335 (2010).

13. Blattner, F. R. *et al.* The complete genome sequence of *Escherichia coli* K-12. *Science* **277**, 1453–1462 (1997).

14. Rendulic, S. *et al.* A predator unmasked: life cycle of *Bdellovibrio bacteriovorus* from a genomic perspective. *Science* **303**, 689–692 (2004).

15. Harding, C. J. *et al.* A lysozyme with altered substrate specificity facilitates prey cell exit by the periplasmic predator *Bdellovibrio bacteriovorus*. *Nature Communications* **11**, 4817 (2020).
