# Supplementary material for: Quantitative proteome of bacterial periplasmic predation by Bdellovibrio bacteriovorus reveals a prey-lytic protease
Source: Commun Biol. 2025 Oct 22;8:1491. doi: 10.1038/s42003-025-09010-x (PMC12546842; doi:10.1038/s42003-025-09010-x)
Supplement: Supplementary file 2 — Description of Additional Supplementary Files [file 42003_2025_9010_MOESM2_ESM.pdf]

Description of Additional Supplementary Files

File name: Supplementary Data 1
Description: A table containing all normalized protein abundances of each protein over a 7-hour period.

File name: Supplementary Data 2
Description: Tables with proteins grouped into the nine clusters of distinct abundance patterns throughout the predatory life cycle.

File name: Supplementary Data 3
Description: Tables containing all the log2 fold change values of *B. bacteriovorus* HD100 proteins between each condition and attack phase.
