# Supplementary material for: Quantitative proteome of bacterial periplasmic predation by Bdellovibrio bacteriovorus reveals a prey-lytic protease
Source: Commun Biol. 2025 Oct 22;8:1491. doi: 10.1038/s42003-025-09010-x (PMC12546842; doi:10.1038/s42003-025-09010-x)
Supplement: Supplementary file 6 — Reporting Summary [file 42003_2025_9010_MOESM6_ESM.pdf]

nature portfolio

Corresponding author(s): Huwiler, S. G.

Last updated by author(s): Sep 10, 2025

Reporting Summary

Nature Portfolio wishes to improve the reproducibility of the work that we publish. This form provides structure for consistency and transparency in reporting. For further information on Nature Portfolio policies, see our Editorial Policies and the Editorial Policy Checklist.

Statistics

For all statistical analyses, confirm that the following items are present in the figure legend, table legend, main text, or Methods section.

n/a	Confirmed	
☐	☒	The exact sample size (*n*) for each experimental group/condition, given as a discrete number and unit of measurement
☐	☒	A statement on whether measurements were taken from distinct samples or whether the same sample was measured repeatedly
☐	☒	The statistical test(s) used AND whether they are one- or two-sided<br>*Only common tests should be described solely by name; describe more complex techniques in the Methods section.*
☐	☒	A description of all covariates tested
☐	☒	A description of any assumptions or corrections, such as tests of normality and adjustment for multiple comparisons
☐	☒	A full description of the statistical parameters including central tendency (e.g. means) or other basic estimates (e.g. regression coefficient) AND variation (e.g. standard deviation) or associated estimates of uncertainty (e.g. confidence intervals)
☐	☒	For null hypothesis testing, the test statistic (e.g. *F*, *t*, *r*) with confidence intervals, effect sizes, degrees of freedom and *P* value noted<br>*Give P values as exact values whenever suitable.*
☒	☐	For Bayesian analysis, information on the choice of priors and Markov chain Monte Carlo settings
☐	☒	For hierarchical and complex designs, identification of the appropriate level for tests and full reporting of outcomes
☒	☐	Estimates of effect sizes (e.g. Cohen's *d*, Pearson's *r*), indicating how they were calculated

Our web collection on statistics for biologists contains articles on many of the points above.

Software and code

Policy information about availability of computer code

Data collection	Experimental data was produced as stated in the article.
Data analysis	Data analysis details are described in the materials and methods section "Quantitative proteome data analysis" of the article. The R codes are made available via Figshare upon publication (DOI: 10.6084/m9.figshare.29533775).

For manuscripts utilizing custom algorithms or software that are central to the research but not yet described in published literature, software must be made available to editors and reviewers. We strongly encourage code deposition in a community repository (e.g. GitHub). See the Nature Portfolio guidelines for submitting code & software for further information.

Data

Policy information about availability of data

All manuscripts must include a data availability statement. This statement should provide the following information, where applicable:

- Accession codes, unique identifiers, or web links for publicly available datasets
- A description of any restrictions on data availability
- For clinical datasets or third party data, please ensure that the statement adheres to our policy

All relevant proteomics data have been deposited to the ProteomeXchange Consortium via the PRoteomics IDEntification Database (PRIDE, http://www.ebi.ac.uk/pride)81 partner repository by EMBL-EBI with the data set identifier PXD056597. All time-lapse microscopy data is available on BioImage Archive (https://www.ebi.ac.uk/bioimage-archive/) with Accession number S-BIAD1580 (https://doi.org/10.6019/S-BIAD1580)82. All further data generated in this study are

available in the associated Supplementary Information file, Supplementary Data 1-3 (xls files). Source material including R codes and the source raw data used are deposited on the Figshare repository (DOI: 10.6084/m9.figshare.29533775). There are no restrictions on data availability such as materials transfer agreements.

Research involving human participants, their data, or biological material

Policy information about studies with human participants or human data. See also policy information about sex, gender (identity/presentation), and sexual orientation and race, ethnicity and racism.

Reporting on sex and gender	N/A
Reporting on race, ethnicity, or other socially relevant groupings	N/A
Population characteristics	N/A
Recruitment	N/A
Ethics oversight	N/A

Note that full information on the approval of the study protocol must also be provided in the manuscript.

Field-specific reporting

Please select the one below that is the best fit for your research. If you are not sure, read the appropriate sections before making your selection.

☒ Life sciences ☐ Behavioural & social sciences ☐ Ecological, evolutionary & environmental sciences

For a reference copy of the document with all sections, see nature.com/documents/nr-reporting-summary-flat.pdf

Life sciences study design

All studies must disclose on these points even when the disclosure is negative.

Sample size	Sample size followed standard in the field. Please refer to the manuscript for details.
Data exclusions	Generally no data was excluded. For details on proteome analysis refer to methods and materials part of the article.
Replication	Replicate size followed the standard in the filed. Please refer to the manuscript, especially the figure legends, for details.
Randomization	No randomization was preformed, as this is not the standard in the field for the experiments in this article.
Blinding	No blinding was preformed, as this is not the standard in the field for the experiments in this article.

Reporting for specific materials, systems and methods

We require information from authors about some types of materials, experimental systems and methods used in many studies. Here, indicate whether each material, system or method listed is relevant to your study. If you are not sure if a list item applies to your research, read the appropriate section before selecting a response.

Materials & experimental systems

n/a	Involved in the study	
☒	☐	Antibodies
☒	☐	Eukaryotic cell lines
☒	☐	Palaeontology and archaeology
☒	☐	Animals and other organisms
☒	☐	Clinical data
☒	☐	Dual use research of concern
☒	☐	Plants

Methods

n/a	Involved in the study	
☒	☐	ChIP-seq
☒	☐	Flow cytometry
☒	☐	MRI-based neuroimaging

Plants

Seed stocks	N/A
Novel plant genotypes	N/A
Authentication	N/A
